# Serum hepcidin may be a novel uremic toxin, which might be related to erythropoietin resistance

**DOI:** 10.1038/s41598-017-04664-y

**Published:** 2017-06-26

**Authors:** Sung Woo Lee, Jeong Min Kim, Hye Jin Lim, Young-Hwan Hwang, Soo Wan Kim, Wookyung Chung, Kook-Hwan Oh, Curie Ahn, Kyu-Beck Lee, Su Ah Sung

**Affiliations:** 1 Department of Internal Medicine, Seoul National University Postgraduate School, Seoul, Korea; 2 Department of Internal Medicine, Eulji Medical center, Eulji University, Seoul, Korea; 3 Department of Internal Medicine, Lohas Geriatric Hospital, Seoul, Korea; 4 Department of Internal Medicine, Gimpo Woori Hospital, Gimpo, Gyeonggi Korea; 5 Department of Internal Medicine, Truewords kidney clinic and institute, Incheon, Korea; 6 Department of Internal Medicine, Chonnam National University Medical School, Chonnam, Korea; 7 Department of Internal Medicine, Gachon University Gil Hospital, Incheon, Korea; 8 Department of Internal Medicine, Seoul National University Hospital, Seoul, Korea; 9 Department of Internal Medicine, Kangbuk Samsung Hospital, Sungkyunkwan University School of Medicine, Seoul, Korea

## Abstract

The clinical importance of serum hepcidin in non-dialysis chronic kidney disease (CKD) patients is unclear. The database of a large-scale multicentre prospective study in Korea of 2238 patients enrolled from 2011–2016 was analysed. After excluding patients with missing serum hepcidin (n = 125) and haemoglobin (n = 23) levels, the study included 2090 non-dialysis CKD patients. Markers of inflammation and iron status were positively associated with serum hepcidin level, regardless of CKD stage. However, estimated glomerular filtration rate was inversely associated with serum hepcidin level, particularly in patients with CKD stages 3b–5 but not in those with CKD stages 1–3a. Use of erythropoiesis-stimulating agents was associated with increased serum hepcidin levels, particularly in patients with CKD stages 3b–5 but not in those with CKD stages 1–3a, and serum hepcidin levels positively correlated with the dose of erythropoiesis-stimulating agent. These findings suggest that serum hepcidin may be a uremic toxin and play an important role in erythropoietin resistance. However, future prospective studies are needed to confirm our results.

## Introduction

Since Tomas Ganz and colleagues described a novel cysteine-rich human peptide in 2001, which they named hepcidin after its origin in the liver (hep-) and its antimicrobial properties (-cidin)^1^, many studies have shown that this peptide plays a key role in iron metabolism^2^. The biological receptor of hepcidin is ferroportin, an iron-exporting transcellular channel located in cells that are sources of iron including enterocytes, macrophages, and hepatocytes^3^. Binding of hepcidin to ferroportin induces the internalization and degradation of ferroportin and disturbs iron efflux from cells to plasma, ultimately reducing serum iron levels and sequestering iron in iron storage sites^3^.

The small size of hepcidin (2.7 kDa) suggests that renal clearance may be a major pathway of elimination and that serum hepcidin levels increase with the progression of chronic kidney disease (CKD)^4, 5^. Although several studies have evaluated factors associated with serum hepcidin levels in non-dialysis CKD patients^4–14^, most previous studies assessed small numbers of patients at single centres, making it unclear whether kidney function is independently associated with serum hepcidin levels. Moreover, the relationship between erythropoiesis-stimulating agents (ESA) and serum hepcidin levels has not been clearly determined in non-dialysis CKD patients^4, 7, 8^. Therefore, this study assessed these relationships in a large number of adults enrolled in the KoreaN cohort study for Outcome in patients With Chronic Kidney Disease (KNOW-CKD).

## Results

The mean age of the 2090 study patients was 53.6 years, and 61.1% were men. Mean estimated glomerular filtration rate (eGFR) was 50.3 ml/min/1.73 m^2^ and the proportions of patients with CKD stages 1, 2, 3a, 3b, 4, and 5 were 11.9%, 18.3%, 18.0%, 21.7%, 23.5%, and 6.6%, respectively. The causes of CKD were diabetic nephropathy in 25.3% of patients, glomerulonephritis in 31.2%, hypertensive nephropathy in 20.1%, and others in 23.4%. ESA and iron supplements were administered to 7.6% and 14.7% of these patients, respectively.

Exploration of baseline characteristics in patients classified by serum hepcidin quartile (Table 1) showed that increased serum hepcidin quartile was associated with increased age and an increased percentage of men, as well as with high rates of hypertension and diabetes. Moreover, increased serum hepcidin quartile was associated with a significant reduction in eGFR and significant increases in white blood cells (WBC) counts and C-reactive protein (CRP). Higher serum hepcidin quartile was also associated with higher rates of anaemia, treatment with ESA and supplemental iron, and higher serum levels of transferrin saturation (TSAT) and ferritin.

**Table 1:** Baseline characteristics of the hepcidin quartile group.

	Serum hepcidin quartile group (n = 2090)				*P*-trend
	1Q (n = 515)	2Q (n = 529)	3Q (n = 521)	4Q (n = 525)	
Age (years)	51.2 ± 12.6	53.8 ± 12.5*	53.8 ± 11.7*	55.4 ± 11.7*	<0.001
Male sex	49.3	62.0*	66.6*	66.1*	<0.001
High income	21.8	25.8	23.5	21.1	0.580
Ever smoking	38.8	45.5*	50.3*^†^	51.6*	<0.001
Hypertension	96.3	97.2	97.9^†^	99.0*	0.003
SBP (mm Hg)	125.6 ± 15.4	127.7 ± 15.2	128.5 ± 16.6*	129.3 ± 17.4*	<0.001
DBP (mm Hg)	76.7 ± 11.1	77.2 ± 10.3	77.3 ± 11.5	76.8 ± 11.7	0.880
Diabetes	28.2	36.9*	36.5*^†^	43.8*^‡^	<0.001
Cause of CKD					
DMN	17.5	23.6*	26.5*^†^	33.5*^‡^	<0.001
GN	39.0	32.3*	29.4*^†^	24.2*	<0.001
HN	18.4	22.3	19.4	20.2	0.777
Others	25.0	21.7	24.8	22.1	0.485
BMI (kg/m^2^)	24.1 ± 3.5	24.6 ± 3.3	25.0 ± 3.5*	24.4 ± 3.3	0.047
Glucose (mmol/l)	5.9 ± 1.9	6.2 ± 2.1	6.3 ± 2.6*	6.2 ± 2.1	0.018
BUN (mmol/l)	8.3 ± 4.8	8.6 ± 4.1	10.1 ± 5.1*^†^	13.3 ± 6.6*^†‡^	<0.001
Creatinine (μmol/l)	128.6 ± 77.6	137.0 ± 72.9	162.4 ± 103.3*^†^	216.9 ± 123.3*^†‡^	<0.001
eGFR (ml/min/1.73 m^2^)	60.8 ± 32.5	55.7 ± 29.6*	48.8 ± 28.5*^†^	36.1 ± 23.9*^†‡^	<0.001
Bilirubin (μmol/l)	11.5 ± 4.7	12.2 ± 5.6	11.6 ± 5.3	10.4 ± 5.0*^†‡^	<0.001
Albumin (g/l)	41.7 ± 3.8	42.2 ± 4.0	41.8 ± 4.3	41.2 ± 4.9^†^	0.013
Cholesterol (mmol/l)	4.6 ± 0.9	4.6 ± 1.0	4.6 ± 1.1	4.4 ± 1.0*^†‡^	0.002
WBC (×10^3^/μL)	6.4 ± 1.9	6.5 ± 1.8	6.7 ± 1.9	6.8 ± 2.1*	0.001
Hemoglobin (g/dl)	13.0 ± 1.9	13.3 ± 1.8	13.0 ± 2.1	11.9 ± 2.0*^†‡^	<0.001
Anaemia	35.9	33.3	42.4*^†^	65.5*^‡^	<0.001
ESA use	2.9	3.8	5.4*^†^	18.4*^‡^	<0.001
Iron supplements	7.4	8.5	13.7*^†^	29.3*^‡^	<0.001
TSAT (%)	28.1 ± 12.4	31.4 ± 11.1*	33.4 ± 11.7*^†^	33.6 ± 12.4*^†^	<0.001
Ferritin (pmol/l)	95.4 (49.3–163.8)	180.7 (120.6–285.9)*	259.1 (170.5–394.3)*^†^	446.7 (282.0–675.2)*^†‡^	<0.001
Hepcidin (ng/ml)	3.9 (2.7–5.3)	9.4 (7.9–11.3)*	18.1 (15.5–21.1)*^†^	38.1 (29.9–56.8)*^†‡^	<0.001
CRP (nmol/l)	4.8 (1.9–12.4)	5.4 (1.9–14.3)	6.7 (2.9–17.1)*^†^	7.6 (2.9–21.9)*^†‡^	<0.001
UPCR (g/g)	0.4 (0.1–1.2)	0.4 (0.1–1.2)	0.5 (0.2–1.8)*^†^	0.7 (0.2–2.1)*^†‡^	<0.001

Q, quartile; SBP, systolic blood pressure; DBP, diastolic blood pressure; CKD, chronic kidney disease; DMN, diabetic nephropathy; GN, glomerulonephritis; HN, hypertensive nephropathy; BMI, body mass index; BUN, blood urea nitrogen; eGFR, estimated glomerular filtration rate; WBC, white blood cells; ESA, erythropoiesis stimulating agents; TSAT, transferrin saturation; CRP, C-reactive protein; UPCR, urine protein-to-creatinine ratio.

Values are expressed as mean ± standard deviation for normally distributed continuous variables, median (interquartile range) for non-normally distributed continuous variables, and percentage for categorical variables. *P*-trend was analyzed by linear-term of one-way ANOVA for normally distributed continuous variables, Jonckheere-Terpstra test for non-normally distributed continuous variables, and a linear-by-linear association for categorical variables. *^,†^, and ^‡^ meant *P* < 0.05 when compared to 1Q, 2Q, and 3Q of serum hepcidin, respectively, by using Bonferroni post-hoc analysis of one-way ANOVA for normally distributed continuous variables, Mann-Whitney U test for non-normally distributed continuous variables w, and chi-square test for categorical variables.

Analysis of haemoglobin levels and markers of iron metabolism and inflammation as a function of CKD stage (Table 2) showed that serum hepcidin levels increased with the progression of CKD stage. Median hepcidin levels in patients with CKD stages 1, 2, 3a, 3b, 4, and 5 were 7.7, 11.5, 11.6, 12.5, 20.5, and 31.6 ng/ml, respectively. Moreover, as CKD stage increased, haemoglobin levels decreased with a statistically significant difference between stage 3a and stage 1. Serum ferritin levels were higher while serum levels of iron and total iron binding capacity (TIBC) were lower as CKD stage increased, with significant differences between stage 2 and stage 1. TSAT also showed a decreasing trend with the progression of CKD stage. WBC count was higher in CKD stage 4 than in stage 1, whereas CRP level was higher in stages 2–5 than in stage 1.

**Table 2:** Trends of hemoglobin, iron metabolism and inflammation by the stage of chronic kidney disease.

	CKD Stage (n = 2090)						*P*-trend
	Stage 1 (n = 248)	Stage 2 (n = 383)	Stage 3a (n = 376)	Stage 3b (n = 454)	Stage 4 (n = 491)	Stage 5 (n = 138)	
eGFR (ml/min/1.73 m^2^)	110.9 ± 20.1	73.2 ± 8.6*	52.2 ± 4.3*^†^	37.3 ± 4.3*^†‡^	23.2 ± 4.4*^†‡§^	11.8 ± 2.4*^†‡§¶^	<0.001
UPCR (g/g)	0.2 (0.1–0.7)	0.2 (0.1–0.7)	0.4 (0.1–1.1)*^†^	0.5 (0.2–1.6)*^†‡^	1.0 (0.3–2.6)*^†‡§^	1.5 (0.7–3.9)*^†‡§¶^	<0.001
Hemoglobin (g/dl)	14.0 ± 1.5	14.1 ± 1.7	13.5 ± 1.9*^†^	12.7 ± 1.8*^†‡^	11.5 ± 1.5*^†‡§^	10.5 ± 1.2*^†‡§¶^	<0.001
TSAT (%)	34.3 ± 14.7	32.8 ± 12.5	32.4 ± 11.6	30.8 ± 10.8*	29.9 ± 11.5*^†‡^	30.6 ± 12.0	<0.001
Serum iron (μmol/l)	19.2 ± 7.5	18.0 ± 6.6*	17.6 ± 6.3*	16.0 ± 5.6*^†‡^	19.2 ± 7.5*^†‡§^	18.0 ± 6.6*^†‡§^	<0.001
Serum TIBC (μmol/l)	57.4 ± 8.7	55.9 ± 8.4*	54.8 ± 8.6*	52.9 ± 9.0*^†‡^	57.4 ± 8.7*^†‡§^	55.9 ± 8.4*^†‡§¶^	<0.001
Ferritin (pmol/l)	170.3 (75.0–349.1)	223.5 (111.6–406.1)*	232.3 (129.2–380.8)*	204.7 (118.1–400.2)*	243.9 (136.0–404.4)*^§^	278.6 (139.1–472.4)*^†‡§^	<0.001
Hepcidin (ng/ml)	7.7 (3.8–14)	11.5 (5.7–18.6)*	11.6 (6.4–20.3)*	12.5 (6.9–25.2)*^†‡^	20.5 (9.9–35.3)*^†‡§^	31.6 (15.6–60.2)*^†‡§¶^	<0.001
WBC (×10^3^/μl)	6.3 ± 1.8	6.5 ± 2.0	6.5 ± 1.9	6.7 ± 1.9	7.0 ± 2.0*^†‡^	6.3 ± 1.8^¶^	0.001
CRP (nmol/l)	3.8 (1.0–10.1)	5.7 (1.9–15.2)*	5.7 (1.9–12.6)*	5.7 (2.9–17.9)*	7.6 (3.3–21.0)*^†‡§^	5.7 (2.4–16.2)*	<0.001

CKD, chronic kidney disease; eGFR, estimated glomerular filtration rate; UPCR, urine protein-to-creatinine ratio; TSAT, transferrin saturation; WBC, white blood cells; CRP, C-reactive protein.

Values are expressed as mean ± standard deviation for normally distributed continuous variables and median (interquartile range) for non-normally distributed continuous variables. *P*-trend was analyzed by a linear-term of one-way ANOVA for normally distributed variables and Jonckheere-Terpstra test for non-normally distributed variables. ^*,†,‡,§^, and ^¶^ meant *P* < 0.05 when compared to CKD stage 1, 2, 3a, 3b, and 4, respectively, by using Bonferroni post-hoc analysis of one-way ANOVA for normally distributed variables and Mann-Whitney U test for non-normally distributed variables.

Multivariable linear regression analysis of factors associated with serum hepcidin levels showed that lower haemoglobin levels and eGFR and higher levels of inflammatory markers (CRP and WBC count) and iron markers (TSAT and ferritin) were independently associated with higher serum hepcidin levels (Table 3). These findings were confirmed in multivariable logistic regression analysis for high serum hepcidin (Supplementary Table S1). CKD stage was independently associated with high serum hepcidin, particularly when comparing CKD stage 3b and higher with stage 1. Subgroup analysis by CKD stage (Table 4) showed that lower haemoglobin level and higher CRP, ferritin, and TSAT were associated with higher serum hepcidin levels in early and advanced CKD. However, decreased eGFR was associated with higher hepcidin in advanced, but not in early, CKD.

**Table 3:** Linear regression analysis for the square root of serum hepcidin level.

	Univariable		Multivariable	
	Beta (95% CI)	*P*	Beta (95% CI)	*P*
Age (years)	0.019 (0.012–0.025)	<0.001	−0.003 (−0.008–0.002)	0.310
Sex (men vs. female)	0.348 (0.183–0.513)	<0.001	−0.125 (−0.300–0.050)	0.160
Income (high vs. non-high)	−0.114 (−0.308–0.081)	0.252	—	—
Ever smoking (yes vs. no)	0.267 (0.106–0.429)	0.001	0.060 (−0.094–0.213)	0.447
SBP (mm Hg)	0.011 (0.006–0.016)	<0.001	0.002 (−0.001–0.006)	0.243
DBP (mm Hg)	−0.002 (−0.010–0.005)	0.536	—	—
BMI (kg/m^2^)	0.014 (−0.009–0.038)	0.236	—	—
Glucose (mmol/l)	0.038 (0.001–0.075)	0.042	−0.015 (−0.042–0.012)	0.284
eGFR (ml/min/1.73 m^2^)	−0.021 (−0.023–−0.018)	<0.001	−0.007 (−0.009–−0.004)	<0.001
Albumin (g/l)	−0.031 (−0.050–−0.012)	0.001	0.032 (0.015–0.049)	<0.001
Cholesterol (mmol/l)	−0.184 (−0.263–−0.105)	<0.001	−0.036 (−0.096–0.024)	0.239
WBC (×10^3^/μl)	0.056 (0.014–0.098)	0.008	0.044 (0.012–0.077)	0.007
CRP (nmol/l)	0.229 (0.170–0.288)	<0.001	0.095 (0.051–0.139)	<0.001
UPCR (g/g)	0.160 (0.108–0.211)	<0.001	0.012 (−0.035–0.06)	0.607
Hemoglobin (g/dl)	−0.234 (−0.272–−0.195)	<0.001	−0.222 (−0.264–−0.181)	<0.001
Ferritin (pmol/l)	1.310 (1.243–1.377)	<0.001	1.226 (1.157–1.295)	<0.001
TSAT (%)	0.027 (0.020–0.034)	<0.001	0.011 (0.006–0.017)	<0.001
ESA use (yes vs. no)	1.951 (1.659–2.244)	<0.001	0.802 (0.563–1.041)	<0.001
Iron supplements (yes vs. no)	1.545 (1.327–1.763)	<0.001	0.306 (0.120–0.492)	0.001
Bilirubin (μmol/l)	−0.045 (−0.061–−0.030)	<0.001	0.012 (−0.002–0.026)	0.082

SBP, systolic blood pressure; DBP, diastolic blood pressure; BMI, body mass index; eGFR, estimated glomerular filtration rate; WBC, white blood cells; CRP, C-reactive protein; UPCR, urine protein-to-creatinine ratio; TSAT, transferrin saturation; ESA, erythropoiesis stimulating agents.

Logarithmic transformations were done for CRP, UPCR and ferritin. In multivariable linear regression analysis, variables with *P* < 0.05 in univariable linear regression analysis were chosen as covariates.

**Table 4:** Subgroup analysis for square root of serum hepcidin level according to CKD stages in multivariable linear regression analysis.

	CKD stage 1–3a		CKD stage 3b–5	
	Beta (95% CI)	*P*	Beta (95% CI)	*P*
Age (years)	0.002 (−0.004–0.009)	0.477	−0.003 (−0.011–0.004)	0.397
Sex (men vs. female)	0.105 (−0.124–0.335)	0.369	−0.260 (−0.519–0.000)	0.050
Ever smoking (yes vs. no)	0.075 (−0.113–0.263)	0.433	0.038 (−0.202–0.277)	0.758
SBP (mm Hg)	−0.002 (−0.007–0.003)	0.460	0.002 (−0.003–0.007)	0.381
Glucose (mmol/l)	−0.010 (−0.051–0.031)	0.641	−0.013 (−0.049–0.023)	0.466
eGFR (ml/min/1.73 m^2^)	−0.002 (−0.005–0.001)	0.279	−0.025 (−0.035–−0.015)	<0.001
Albumin (g/l)	0.049 (0.026–0.072)	<0.001	0.025 (0.001–0.050)	0.045
Cholesterol (mmol/l)	0.023 (−0.056–0.103)	0.569	−0.075 (−0.160–0.010)	0.085
WBC (×10^3^/μl)	0.033 (−0.009–0.076)	0.125	0.049 (0.003–0.096)	0.037
CRP (nmol/l)	0.079 (0.021–0.136)	0.008	0.119 (0.055–0.182)	<0.001
UPCR (g/g)	−0.009 (−0.066–0.047)	0.745	0.019 (−0.059–0.097)	0.632
Hemoglobin (g/dl)	−0.165 (−0.221–−0.109)	<0.001	−0.187 (−0.250–−0.124)	<0.001
Ferritin (pmol/l)	0.939 (0.847–1.031)	<0.001	1.460 (1.359–1.562)	<0.001
TSAT (%)	0.010 (0.004–0.017)	0.002	0.010 (0.002–0.019)	0.015
ESA use (yes vs. no)	−1.366 (−2.944–0.213)	0.090	0.655 (0.386–0.924)	<0.001
Iron supplements (yes vs. no)	0.647 (0.317–0.978)	<0.001	0.125 (−0.104–0.354)	0.284
Bilirubin (μmol/l)	0.013 (−0.002–0.028)	0.093	0.017 (−0.008–0.043)	0.178

SBP, systolic blood pressure; eGFR, estimated glomerular filtration rate; WBC, white blood cells; CRP, C-reactive protein; UPCR, urine protein-to-creatinine ratio; TSAT, transferrin saturation; ESA, erythropoiesis stimulating agents.

Logarithmic transformations were done for CRP, UPCR and ferritin. Variables with *P* < 0.05 in univariable linear regression analysis were chosen as covariates in multivariable linear regression analysis.

**Figure 1:**
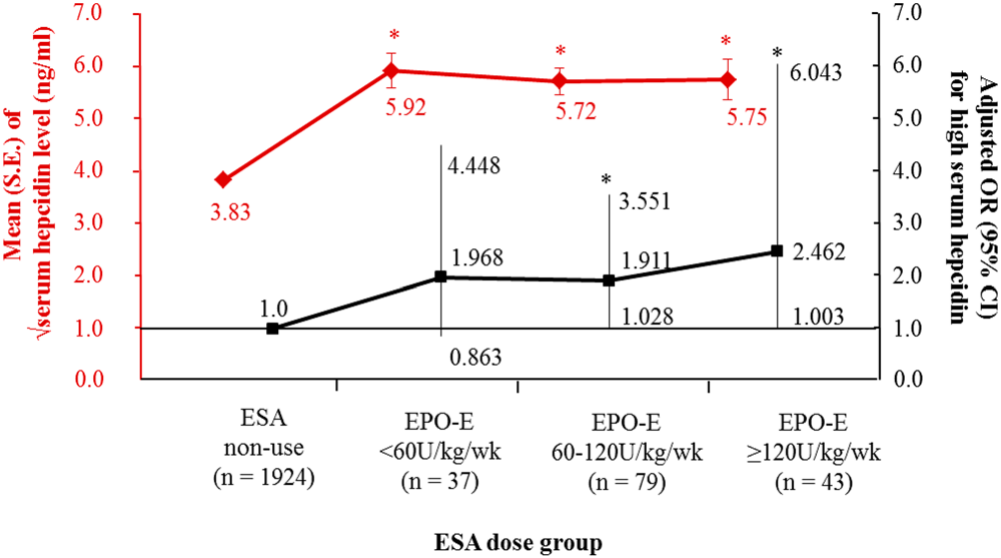
Dose relationship between erythropoietin stimulating agents (ESA) usage and serum hepcidin level. S.E., standard error; EPO-E, epoetin-equivalent. *Meant *P* < 0.05 when compared to ESA non-use group. Adjusted odds ratio (OR) and it confidence interval (CI) for high serum hepcidin were calculated by using multivariable logistic regression entering age, sex, ever smoking, hypertension, diabetes, stage of chronic kidney disease, hemoglobin, transferrin saturation, iron supplements, white blood cells, C-reactive protein, urine protein-to-creatinine ratio, albumin, cholesterol and bilirubin as covariates.

**Figure 2:**
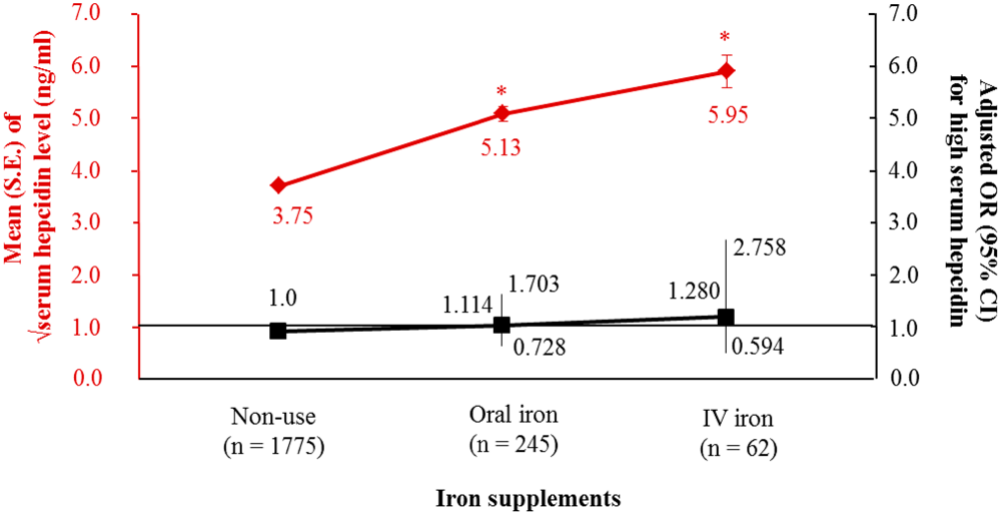
Dose relationship between route of iron supplements and serum hepcidin level. S.E., standard error; IV, intravenous. *Meant *P* < 0.05 when compared to non-use group. Adjusted odds ratio (OR) and it confidence interval (CI) for high serum hepcidin were calculated by using multivariable logistic regression entering age, sex, ever smoking, hypertension, diabetes, stage of chronic kidney disease, hemoglobin, transferrin saturation, iron supplements, white blood cells, C-reactive protein, urine protein-to-creatinine ratio, albumin, cholesterol and bilirubin as covariates.

We found that both ESA treatment and iron supplementation were associated with higher serum hepcidin levels (Table 3). Subgroup analysis by CKD stage showed that serum hepcidin levels were associated with iron supplementation in patients with early CKD and with ESA treatment in patients with advanced CKD (Table 4). Multivariable logistic regression analysis showed that ESA treatment, but not iron supplementation, was associated with high serum hepcidin levels (Supplementary Table S1). Assessment of the relationships of ESA dose and iron supplement routes with serum hepcidin showed that increased ESA dose was associated with a significant increase in the square root of serum hepcidin levels (Fig. 1). Multivariable logistic analysis showed that patients taking 60–120 and ≥120 IU/kg/week ESA showed 1.9-fold (*P* = 0.041) and 2.5-fold (*P* = 0.049) higher odds for high serum hepcidin, respectively, than patients not taking ESA. Although the square root of serum hepcidin levels progressively and significantly increased from patients not treated with iron supplements to those taking oral iron to those taking intravenous iron, multivariable analysis showed that iron supplements, regardless of route, were not associated with high serum hepcidin (Fig. 2).

## Discussion

Several studies to date have assessed the clinical importance of serum hepcidin in non-dialysis CKD patients (Supplementary Table S2)^4–14^. Although ferritin was found to be an independent predictor of serum hepcidin levels, a finding confirmed in our study, the relationship between eGFR and hepcidin is unclear. Because most previous studies included small numbers of patients at single centres, this study analysed the association between eGFR and serum hepcidin using baseline data from a large-scale prospective multicentre cohort in Korea.

We found that serum hepcidin levels directly correlated with CKD stage and inversely correlated with eGFR. These findings were confirmed in multivariable logistic regression analysis, suggesting a possible non-linear association between kidney function and serum hepcidin levels as the odds for high serum hepcidin were statistically evident from CKD stage 3b compared with CKD stage 1. Subgroup analysis by CKD stage showed that factors associated with anaemia (haemoglobin levels), iron metabolism (TSAT and ferritin levels), and inflammation (CRP levels) were associated with serum hepcidin levels, regardless of CKD stage. In contrast, eGFR was associated with serum hepcidin level only in patients with advanced (stages 3b–5), but not in early (stage 1–3a), CKD, suggesting that the pathogenesis of elevated serum hepcidin may differ in advanced and early CKD and that decreased renal clearance may significantly contribute to elevated serum hepcidin levels in advanced CKD. By definition^15^, therefore, hepcidin can be classified as a novel uremic toxin.

We found that studies, including ours, reporting that eGFR was an independent predictor of serum hepcidin levels have commonly measured hepcidin using competitive enzyme-linked immunosorbent assay (cELISA)^4, 5, 10–12^, whereas studies suggesting that eGFR was a confounding factor for serum hepcidin have measured hepcidin by mass spectrometry (MS)^6–9^ (Supplementary Table S2). These results suggest that the lack of agreement between studies assessing eGFR as a predictor of serum hepcidin level may be attributed to differences in the assays used to measure hepcidin levels. Since hepcidin was initially discovered using mass spectrum analysis^1^, MS has been a mainstay of hepcidin measurement. However, accessibility to MS is limited because it requires specialists and complex equipment^16^. As a more convenient assay, cELISA was developed by the same group who discovered hepcidin^17^. Subsequent studies have validated the good correlation between results obtained by cELISA and MS^18^. Unlike MS, however, cELISA is poor at differentiating hepcidin isoforms^18, 19^. In addition to its bioactive isoform, hepcidin-25, hepcidin can exist in other isoforms, including hepcidin-20, 22, and 24^20^. The percentage of these other hepcidin isoforms is higher in CKD patients than in controls, representing as much as 20% of total serum hepcidin in the former^19^. If the association between hepcidin and eGFR is affected by hepcidin isoforms^8^, then hepcidin measured by cELISA and by MS may exhibit different clinical characteristics.

This study also found that treatment with ESA was significantly associated with increased serum hepcidin levels, particularly in patients with advanced CKD. Moreover, we observed a positive relationship between serum hepcidin and ESA dose, independent of haemoglobin level. These results differ from those of a previous study which found an inverse correlation between serum hepcidin level and ESA dose in 94 haemodialysis patients^4^. That study also reported that seven ESA-naïve non-dialysis CKD patients treated with ESA for 4 weeks showed decreased serum hepcidin levels^4^. A randomized controlled study of 33 non-dialysis CKD patients showed that serum hepcidin levels were decreased after 2 weeks of treatment with ESA, suggesting that the ESA-associated change in hepcidin level predicted an early and long-term bone marrow response^21^. These previous studies, however, found that ESA affected serum hepcidin levels, not vice versa. Our cross-sectional study in a large population suggests that hepcidin level can affect ESA requirements in patients with CKD. Patients with high hepcidin levels may have ESA-resistant anaemia because of the low availability of iron, despite taking iron supplements, and may require high ESA doses. In this study, ESA users had higher rates of iron supplementation and iron sequestration (higher serum ferritin despite similar TSAT) than non-users (Supplementary Table S3). This hypothesis can be supported by a recent clinical trial which found that ESA response was improved by the manipulation of serum hepcidin with an oral inhibitor of hypoxia inducible factor^22^.

The current study also found that iron supplementation was associated with increased serum hepcidin levels, in agreement with previous studies. Chand *et al*. suggested that serum hepcidin levels in 129 non-dialysis CKD patients increased after 6 weeks of iron supplementation^7^. Gaillard *et al*. also reported that serum hepcidin levels in 61 non-dialysis CKD patients increased after 52 weeks of iron therapy, administered intravenously or orally^9^. We observed a positive association between iron supplementation and serum hepcidin levels in patients with early, but not advanced, CKD. This may explain the lack of association between iron supplements and high (≥25.1 ng/ml) serum hepcidin, given that serum hepcidin levels are about two-fold higher in advanced than in early CKD.

This study had several limitations, including its cross-sectional design, thereby preventing a determination of the cause-effect relationships related to serum hepcidin levels. Careful interpretation of these results is therefore required, especially when assessing the effect of ESA on serum hepcidin. However, our study cohort was much larger than those of previous cross-sectional studies (Supplementary Table S2), enabling analysis of the association between serum hepcidin and eGFR according to CKD stage. Second, we measured serum hepcidin by cELISA, which cannot distinguish among hepcidin isoforms. To our knowledge, no current method of measuring hepcidin can be considered the gold standard^16^. Immunoassays using chromatography^23^ and MS cannot determine the actual level of hepcidin-25 because of pre-analytic peptide loss, and the results of these assays are also semi-quantitative^16^. Additional studies are thus required to identify more accurate methods of measuring hepcidin level and to determine the significance of hepcidin isoforms other than hepcidin-25^24^. Finally, although our study included a large number of patients, they were from a single country and represented a single ethnic group, thereby limiting the generalizability of our results.

In conclusion, decreased kidney function was associated with increased serum hepcidin levels, especially in patients with advanced CKD. Decreased haemoglobin levels and higher levels of iron markers were also associated with higher serum hepcidin levels. Iron supplementation was positively correlated with serum hepcidin levels, especially in patients with early CKD. The higher hepcidin level in ESA users, particularly in those with advanced CKD, suggests that hepcidin is a key peptide in ESA resistance. These results may prompt future longitudinal studies on the clinical significance of serum hepcidin, measured by cELISA, in non-dialysis CKD patients.

## Methods

### Participants

The KNOW-CKD is a multicentre prospective cohort study in Korea of 2238 patients with non-dialysis CKD stages 1–5 enrolled from February 2011 through January 2016. The detailed design and methods of the KNOW-CKD have been previously published (NCT01630486 at http://www.clinicaltrials.gov)^25^. The protocol of the KNOW-CKD adhered to the principles of the Declaration of Helsinki and was approved by the Institutional Review Board at each participating hospital including Seoul National University Hospital, Yonsei University Severance Hospital, Kangbuk Samsung Medical Center, Seoul St. Mary’s Hospital, Gil Hospital, Eulji Medical Center, Chonnam National University Hospital, and Pusan Paik Hospital. Written informed consent was obtained from all subjects. eGFR was calculated using the equation of Modification of Diet in Renal Disease study formula^26^. CKD and its stages were defined using the Kidney Disease Improving Global Outcomes 2012 guidelines^27^.

Of the 2238 cohort subjects, 148 were excluded, including 125 with missing serum hepcidin levels and 23 with missing hemoglobin levels. This study therefore included 2090 patients.

### Serum hepcidin measurement

Serum hepcidin levels were measured at a central laboratory by cELISA using EIA5258 kits (DRG Diagnostics, Marburg, Germany), according to the manufacturer’s instructions. The intra- and inter-assay coefficients of variation ranged from 2.1–9.9% and from 11.5–14.6%, respectively. The detectable maximum level was 80 ng/ml, with higher levels recorded as 80 ng/ml.

### Definitions

Clinical data, including detailed demographic information and baseline laboratory results, were extracted from the electronic data management system (PhactaX). Hypertension was defined as physician diagnosis, systolic blood pressure (BP) ≥140 mm Hg or diastolic BP ≥90 mmHg, or treatment with anti-hypertensive drugs. Diabetes was defined as physician diagnosis, fasting glucose ≥126 mg/dl, or treatment with insulin or oral anti-diabetic drugs. High income was defined as a monthly household income of more than 4.5 million won (approximately 4000 US dollars). Ever smoking was defined as past or current smoking. Body mass index was calculated as weight (kg) per square of height (m^2^). Anaemia was defined as haemoglobin <13.0 g/dl in men and <12.0 g/dl in women^28^. TSAT (%) was calculated as serum iron × 100/TIBC. Dose of ESA was measured as the weight-normalized epoetin-equivalent (IU/kg/week), with 1 µg of darbepoetin alpha converted to 331 units of epoetin^29^. Continuous erythropoietin receptor activator doses of 50 µg/month, 75 µg/month, 100 µg/month, and 150 µg/month were converted to epoetin equivalents of 3000 IU/week, 4000 IU/week, 6000 IU/week, and 8000 IU/week, respectively^30^. Serum hepcidin levels were divided into quartiles, with the first, second, third, and fourth quartiles defined as <6.6 ng/ml, 6.6–13.4 ng/ml, 13.4–25.1 ng/ml and ≥25.1 ng/ml, respectively. The fourth quartile was defined as high serum hepcidin. Patients were also sub-grouped by CKD stage into early (stage 1–3a) and advanced (stage 3b–5) CKD.

### Statistical analysis

The distributions of continuous variables were evaluated using histograms and Q-Q plots. Four variables, hepcidin, ferritin, CRP, and urine protein-to-creatinine ratio (UPCR) were not normally distributed. Normally distributed continuous variables were expressed as mean ± standard deviation, non-normally distributed continuous variables as median (interquartile range), and categorical variables as percentages. *P*-trend was analysed for normally distributed continuous variables by a linear-term of one-way analysis of variance (ANOVA), for non-normally distributed continuous variables by Jonckheere-Terpstra tests, and for categorical variables by a linear-by-linear association. Differences were analysed by Bonferroni post-hoc analysis of one-way ANOVA for normally distributed continuous variables, Mann-Whitney U tests for non-normally distributed continuous variables, and chi-square tests for categorical variables. The square roots of serum hepcidin levels and the logarithm of CRP, UPCR, and ferritin values were utilized in linear regression analysis. Odds ratio (OR) and 95% confidence interval (CI) were calculated by logistic regression analysis. A *P* value < 0.05 was considered statistically significant. In multivariable analysis, variables with statistical significance on univariable analyses were chosen as covariates using the enter method. All analyses were performed using SPSS version 22 software (IBM Corp. released 2013, Armonk, NY: IBM Corp).

## Electronic supplementary material

Supplemental Tables
