# Supplementary material for: Serum hepcidin may be a novel uremic toxin, which might be related to erythropoietin resistance
Source: Sci Rep. 2017 Jun 26;7:4260. doi: 10.1038/s41598-017-04664-y (PMC5484693; doi:10.1038/s41598-017-04664-y)
Supplement: Supplementary file 1 — Supplemental Tables [file 41598_2017_4664_MOESM1_ESM.doc]

**Supplementary material**

**Serum hepcidin may be a novel uremic toxin, which might be related to erythropoietin resistance**

Sung Woo Lee^1,2^, Jeong Min Kim^3^, Hye Jin Lim^4^, Young-Hwan Hwang^5^, Soo Wan Kim^6^, Wookyung Chung^7^, Kook-Hwan Oh^8^, Curie Ahn^8^, Kyu-Beck Lee^9^, and *Su Ah Sung^2^

*^1^Department of Internal Medicine, Seoul National University Postgraduate School, Seoul, Korea*

*^2^Department of Internal Medicine, Eulji Medical center, Eulji University, Seoul, Korea*

*^3^Department of Internal Medicine, Lohas Geriatric Hospital, Seoul, Korea*

*^4^Department of Internal Medicine, Gimpo Woori Hospital, Gimpo, Gyeonggi, Korea*

*^5^Department of Internal Medicine, Truewords kidney clinic and institute, Incheon, Korea*

*^6^Department of Internal Medicine, Chonnam National University Medical School, Chonnam, Korea*

*^7^Department of Internal Medicine, Gachon University Gil Hospital, Incheon, Korea*

*^8^Department of Internal Medicine, Seoul National University Hospital, Seoul, Korea*

*^9^Department of Internal Medicine, Kangbuk Samsung Hospital, Sungkyunkwan University School of Medicine, Seoul, Korea*

***Correspondence to: Su Ah Sung, MD**

e-mail : soi@eulji.ac.kr

Tel) 82-2-970-8205

Fax) 82-2-971-8212

Mailing address) Division of Nephrology, Department of Internal Medicine, Eulji Medical Center, 68, Hangeulbiseok-ro, Nowon-gu, Seoul, 01735, Korea

**Running headline:** Hepcidin in patients with CKD

Table S1. Logistic regression analysis for the high serum hepcidin

|  | Univariable | | Multivariable | |
| --- | --- | --- | --- | --- |
|  | OR (95% CI) | *P* | OR (95% CI) | *P* |
| Age (year) | 1.017 (1.009–1.026) | <0.001 | 1.004 (0.992–1.016) | 0.541 |
| Sex (men vs. women) | 1.335 (1.085–1.641) | 0.006 | 1.122 (0.742–1.697) | 0.585 |
| Income (high vs. non-high) | 0.859 (0.674–1.095) | 0.220 | - | - |
| Ever smoking (yes vs. no) | 1.310 (1.075–1.597) | 0.008 | 1.119 (0.782–1.600) | 0.540 |
| Hypertension (yes vs. no) | 3.079 (1.216–7.798) | 0.018 | 1.205 (0.356–4.078) | 0.764 |
| Diabetes (yes vs. no) | 1.521 (1.243–1.861) | <0.001 | 0.854 (0.622–1.174) | 0.332 |
| BMI (kg/m^2^) | 0.989 (0.960–1.019) | 0.462 | - | - |
| CKD stages (vs. stage 1) |  | <0.001 |  | <0.001 |
| Stage 2 | 1.759 (1.044–2.964) | 0.034 | 1.621 (0.869–3.024) | 0.129 |
| Stage 3a | 1.951 (1.163–3.273) | 0.011 | 1.250 (0.650–2.404) | 0.504 |
| Stage 3b | 3.444 (2.118–5.602) | <0.001 | 2.293 (1.208–4.354) | 0.011 |
| Stage 4 | 6.429 (4.002–10.327) | <0.001 | 3.639 (1.881–7.038) | <0.001 |
| Stage 5 | 15.980 (9.170–27.847) | <0.001 | 6.958 (3.163–15.305) | <0.001 |
| Hemoglobin (g/dl) | 0.732 (0.694–0.773) | <0.001 | 0.743 (0.670–0.824) | <0.001 |
| TSAT (%) | 1.017 (1.009–1.025) | <0.001 | 1.013 (1.000–1.027) | 0.044 |
| Ferritin (pmol/l) | 1.005 (1.004–1.006) | <0.001 | 1.005 (1.005–1.006) | <0.001 |
| ESA use (yes vs. no) | 5.375 (3.843–7.517) | <0.001 | 2.031 (1.248–3.305) | 0.004 |
| Iron supplements (yes vs. no) | 3.786 (2.944–4.867) | <0.001 | 1.142 (0.767–1.701) | 0.512 |
| WBC (1000/μL) | 1.061 (1.008–1.116) | 0.022 | 1.088 (1.010–1.171) | 0.026 |
| CRP (nmol/l) | 1.005 (1.003–1.007) | <0.001 | 1.003 (1.001–1.006) | 0.014 |
| UPCR (g/g) | 1.111 (1.064–1.159) | <0.001 | 1.010 (0.929–1.098) | 0.816 |
| Albumin (g/l) | 0.961 (0.940–0.982) | <0.001 | 1.041 (0.996–1.089) | 0.074 |
| Cholesterol (mmol/l) | 0.797 (0.719–0.884) | <0.001 | 0.948 (0.820–1.096) | 0.472 |
| Bilirubin (μmol/l) | 0.941 (0.921–0.963) | <0.001 | 1.034 (1.001–1.068) | 0.043 |

OR, odds ratio; CI, confidence interval; BMI, body mass index; CKD, chronic kidney disease; TSAT, transferrin saturation; ESA, erythropoiesis stimulating agents; WBC, white blood cells; CRP, C-reactive protein; UPCR, urine protein-to-creatinine ratio.

In multivariable logistic regression analysis, variables with *P* < 0.05 in univariable logistic regression were chosen as covariates.

Table S2. Studies regarding serum hepcidin in non-dialysis adult chronic kidney disease patients

| **Authors** | **Nation** | **Center** | **Sample size* (control)** | **Measurement of hepcidin** | **Association with serum hepcidin in non-dialysis CKD patients*** | | | |
| --- | --- | --- | --- | --- | --- | --- | --- | --- |
|  |  |  |  |  | **eGFR** | **Ferritin** | **TSAT** | **CRP** |
| Uehata (2012)^6^ | Japan | Single | 505 (No) | MS | NO | YES | Not evaluated | NO |
| Chand (2015)^7^ | UK | Single | 129 (No) | MS | NO | YES | yes | YES |
| Peters (2010)^8^ | Netherlands | Single | 83 (24) | MS | NO | YES | no | no |
| Gaillard (2016)^9^ | Europe | Multi | 61 (No) | MS with UPLC | no | yes | yes | Not evaluated |
| Mercadel (2014)^10^ | France | Multi | 199 (No) | cELISA | YES | YES | YES | Not evaluated |
| Troutt (2013)^11^ | US | Single | 103 (100) | cELISA | yes | yes | Not evaluated | Not evaluated |
| Yang (2014)^12^ | China | Single | 90 (40) | cELISA | yes | Not evaluated | Not evaluated | no |
| Lukaszyk (2015)^13^ | Poland | Single | 69 (No) | cELISA | NO | YES | Not evaluated | NO |
| Ashby (2009)^4^ | UK | Single | 44 (64) | cELISA | yes | yes | Not evaluated | no |
| Zaritsky (2009)^5^ | US | Single | 32 (24) | cELISA | YES | YES | YES | YES |
| Drakou (2016)^14^ | Greece | Single | 78 (No) | cELISA | no | yes | Not evaluated | no |

CKD, chronic kidney disease; eGFR, estimated glomerular filtration rate; TSAT, transferrin saturation; CRP, C-reactive protein; cELISA, competitive enzyme-linked immunosorbent assay; MS, mass spectometry; UPLC, ultra-high pressure liquid chromatography; US, united states; UK, united kingdom. Sample size counted only in non-dialysis CKD patients. *Capital character means results from multivariable analysis, while lowercase character signifies results from univariable analysis.

Table S3. Clinical characteristics according to the usage of erythropoietin stimulating agents

|  | **ESA non-user (n = 1924)** | **ESA user (n = 159)** | ***P*** |
| --- | --- | --- | --- |
| Age (years) | 53.3 ± 12.3 | 57.1 ± 11.1 | <0.001 |
| Male sex | 62.7 | 40.3 | <0.001 |
| High income | 23.7 | 13.5 | 0.003 |
| Ever smoking | 47.5 | 34.0 | 0.001 |
| Hypertension | 97.6 | 98.1 | 1.000 |
| Diabetes | 34.8 | 56.6 | <0.001 |
| BMI (kg/m^2^) | 24.6 ± 3.4 | 23.5 ± 3.0 | <0.001 |
| BUN (mmol/l) | 9.5 ± 5.0 | 18.0 ± 6.9 | <0.001 |
| Creatinine (μmol/l) | 149.9 ± 88.5 | 299.9 ± 148.7 | <0.001 |
| eGFR (ml/min/1.73m^2^) | 52.9 ± 30.0 | 19.6 ± 9.0 | <0.001 |
| WBC (×10^3^/μL) | 6.6 ± 1.9 | 6.4 ± 1.8 | 0.131 |
| Hemoglobin (g/dl) | 13.0 ± 2.0 | 10.6 ± 1.4 | <0.001 |
| Anemia | 40.6 | 89.9 | <0.001 |
| Hemoglobin <10g/dl | 5.2 | 32.7 | <0.001 |
| Iron supplements | 11.1 | 58.9 | <0.001 |
| TSAT (%) | 31.8 ± 11.9 | 30.3 ± 14.2 | 0.153 |
| Ferritin (pmol/l) | 217.1 (117.5–385.6) | 273.8 (140.1–514.8) | <0.001 |
| Hepcidin (ng/ml) | 12.5 (6.4–23.1) | 33.3 (16.9–62.1) | <0.001 |
| CRP (nmol/l) | 5.7 (2.3–15.7) | 4.8 (1.9–14.3) | 0.087 |
| UPCR (g/g) | 0.5 (0.1–1.4) | 1.2 (0.4–2.9) | <0.001 |

BMI, body mass index; BUN, blood urea nitrogen; eGFR, estimated glomerular filtration rate; WBC, white blood cells; ESA, erythropoiesis stimulating agents; TSAT, transferrin saturation; CRP, C-reactive protein; UPCR, urine protein-to-creatinine ratio.

Values are expressed as mean ± standard deviation for normally distributed continuous variables, median (interquartile range) for non-normally distributed continuous variables, and percentage for categorical variables. Difference was analyzed by t test or Mann-Whitney U test for continuous variables, and chi-square test for categorical variables.
